# Sleep Deprivation‐Induced Anxiety Alleviated by Oral Administration of 4‐Aminopyridine in Male Mice

**DOI:** 10.1002/brb3.70382

**Published:** 2025-03-09

**Authors:** Ehsan Hosseini

**Affiliations:** ^1^ Division of Physiology, Department of Basic Science, Faculty of Veterinary Medicine Urmia University Urmia Iran

**Keywords:** 4‐Aminopyridine, anxiety, hippocampus, mice, oxidative stress, sleep deprivation

## Abstract

**Purpose:**

Insufficient sleep and insomnia are common issues associated with modern lifestyles that often contribute to the development of mental health disorders. 4‐aminopyridine (4‐AP), a voltage‐gated potassium (Kv) channel antagonist, is commonly used in the treatment of multiple sclerosis (MS). It has been shown to improve nerve conduction velocity, strengthen myelin, and increase axonal area after injury. In addition, 4‐AP has been reported to reduce behavioral disorders, including depression. The aim of this study was to investigate the effects of 4‐AP on anxiety‐like behavior in mice subjected to rapid eye movement (REM) sleep deprivation.

**Methods:**

Fifty male mice were randomly divided into five groups: control, normal saline (NS) (receiving normal saline via gavage), AP‐0.25, AP‐0.5, and AP‐1 (receiving daily doses of 0.25, 0.5, and 1 mg/kg of 4‐AP, respectively by gavage). All groups except the control group underwent SD for five consecutive days. The animals' locomotion and anxiety‐like behavior were assessed using the open field and elevated plus maze tests. After behavioral testing, N‐methyl‐D‐aspartate receptor (NMDA‐R), α‐amino‐3‐hydroxy‐5‐methyl‐4‐isoxazolepropionic acid receptor (AMPA‐R), and tumor necrosis factor (TNF‐α) were measured by western blotting, and also malondialdehyde (MDA) and total antioxidant capacity (TAC) were analyzed by ELISA in the hippocampus.

**Finding:**

AP‐1 significantly reduced the levels of anxiety‐like behavior compared to the NS group in both tests. In AP‐1, a significant decrease in the levels of NMDA‐R, AMPA‐R, TNF‐α, and MDA was observed. While these levels were increased in the NS group. In addition, AP‐1 showed a higher level of TAC compared to the NS group, indicating an increase in antioxidant levels.

**Conclusion:**

4‐AP may be effective in reducing anxiety‐like behavior in sleep‐deprived mice by modifying the levels of NMDA‐R, AMPA‐R, and TNF‐α, while simultaneously reducing oxidative stress induced by sleep deprivation in the hippocampus.

## Graphical abstract

4‐AP may be effective in attenuating anxiety‐like behavior in sleep‐deprived mice by modifying the levels of NMDA‐R and AMPA‐R and TNF‐α and simultaneously reducing oxidative stress in the hippocampus.

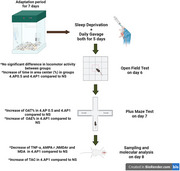

## 1 Introduction

Modern human life is a direct result of advances in scientific research; however, this advancement is accompanied by numerous challenges in daily routine. Among these, stress stands out as a major concern, which is recognized as a potential risk factor for the development of various psychological disorders, such as depression and anxiety (Rojas‐Carvajal et al. 2022). Insomnia, a widespread issue in contemporary society, is a major source of stress (Sanches et al. 2015). Research has shown that anxiety caused by insomnia can lead to a range of psychological disorders, including depression, as identified in previous studies (Neckelmann et al. 2007; Wittchen et al. 2000). Moreover, cognitive impairment has also been identified as a consequence of insomnia‐related anxiety (Robinson et al. 2013; Attwood et al. 2017).

Sleep deprivation (SD) in mice has been well documented to lead to impaired social behaviors (Saré et al. 2019), including memory deficits (Patti et al. 2010), increased aggression (Pires et al. 2018), and decreased sexual activity (Saré et al. 2019). Furthermore, there is a strong association between anxiety‐like behaviors and SD.

In recent years, a wide range of pharmacological agents have been developed to address anxiety disorders. These include benzodiazepines and selective serotonin reuptake inhibitors (SSRIs), which are associated with significant side effects (Uzun et al. 2010; Edinoff et al. 2021).

In research laboratories, 4‐aminopyridine (4‐AP) is widely used as a pharmacological tool for studying potassium conductance in physiology and biophysics. It acts as a nonselective blocker of the Kv1 family of voltage‐gated K^+^ channels (Taglialatela and Luisi 2009). Although 4‐AP has been shown to initiate the activity of voltage‐gated Ca^2^+ channels independently of its effects on voltage‐gated K^+^ channels (Wu et al. 2009). It is clinically used in the treatment of Lambert–Eaton myasthenia gravis and multiple sclerosis (MS). Its mechanism of action involves blocking voltage‐gated potassium channels, which prolongs action potentials and increases neurotransmitter release at the neuromuscular junction (Judge and Bever 2006). It has been shown to counteract the toxicity of saxitoxin and tetrodotoxin in tissue and animal studies (Chang et al. 1997; Chen et al. 1996). In cases of calcium channel blocker overdose in humans, 4‐AP has been shown to effectively increase cytosolic Ca^2+^ concentrations independently of calcium channels (van der Voort et al. 2016). As mentioned, the drug 4‐AP is considered a broad and nonselective inhibitor of “voltage‐gated potassium” (KV) channels (Khammy et al. 2018). It is commonly used as a symptomatic treatment for various neurological disorders, including MS. Previous studies have shown that 4‐AP has neuroprotective effects and improves deficits in motor function (Dietrich et al. 2021), cognitive function (Broicher et al. 2018), and anxiety‐related behaviors (Marzal‐Alfaro et al. 2018). However, it is important to emphasize that these findings may not be generalizable to healthy subjects. Studies have reported that intraperitoneal injection of 4‐AP at a dose of 10 mg/kg induces significant seizure activity in mice (Fedor et al. 2020). In contrast, the present study used very low oral doses of 0.25, 0.5, and 1 mg/kg. However, studies have shown that dose‐dependent toxicity in humans following 4‐AP administration is common, with reported adverse effects including dizziness, insomnia, paresthesia, asthenia, headache, tremor, delirium, choreoathetosis, and seizures (Schwam 2009).

Rapid eye movement (REM) SD has been shown to induce anxiety‐like behavior (Mathangi et al. 2012), and 4‐AP has shown potential in reducing anxiety (Marzal‐Alfaro et al. 2018). Given that SD is closely associated with increased levels of anxiety (Yoshiike et al. 2020; Moffitt et al. 2007), this study aimed to investigate the effects of 4‐AP on anxiety‐like behavior in REM sleep‐deprived mice.

The hippocampus, a complex brain structure located deep in the temporal lobe, plays an important role in learning and memory processes (Anand and Dhikav 2012). Its main function is to consolidate short‐term memories and facilitate their transfer to long‐term storage in the brain. In addition, the hippocampus plays an important role in mood regulation, particularly in modulating anxiety‐related behaviors (Ghasemi et al. 2022; Engin and Treit 2007). While the hippocampus, amygdala, and prefrontal cortex are all critical brain regions involved in anxiety regulation, the hippocampus was selected for this study because of its central role in regulating anxiety‐related behavior. This is supported by the involvement of the hippocampal‐prefrontal cortex pathway (Zhang et al. 2022), the hippocampal‐amygdala projections (Bakir 2015), and the hippocampal‐hypothalamic circuit (Jimenez et al. 2018) in anxiety modulation. Furthermore, it is important to highlight the key role of the hippocampus in anxiety‐like behaviors induced by SD in mice (Yin et al. 2017). The NMDA receptor (NMDA‐R) is a glutamate‐binding receptor, and glutamate is the primary excitatory neurotransmitter in the human brain (Jewett and Thapa 2022). Research has shown that activation of NMDA‐R in the hippocampus can induce anxiety‐like responses in rodents (Barkus et al. 2010; Grillon et al. 2003). Similarly, AMPA receptors (AMPA‐R) play an important role in facilitating glutamatergic neurotransmission in the brain (Gan et al. 2015), and their activation has also been associated with the development of anxiety‐like behaviors (Kapus et al. 2008; Andreasen et al. 2015). Tumor TNF‐α, a key regulator of inflammatory responses, is involved in the pathogenesis of a variety of inflammatory and autoimmune disorders (Jang et al. 2021). Furthermore, TNF‐α has been associated with increased anxiety‐like behaviors (Fourrier et al. 2019; Kemp et al. 2022; Fourrier et al. 2019). Notably, studies have shown that TNF‐α can activate both NMDA‐R and AMPA‐R in the brain, further emphasizing its role in anxiety‐related mechanisms (Olmos and Lladó 2014; Jara et al. 2007). Given the established role of these factors in anxiety‐related behaviors, our study was guided by two key questions: First, based on findings from human studies showing that 4‐AP effectively reduces anxiety in patients with MS (Marzal‐Alfaro et al. 2018), could 4‐AP also exhibit anxiolytic effects in sleep‐deprived mice? Second, could these potential anxiolytic effects be associated with changes in the expression levels of AMPA‐R, NMDA‐R, and TNF‐α in the hippocampus? Oxidative stress, characterized by an imbalance between free radical production and the body's antioxidant defenses, can have detrimental effects on various organs and tissues. In the brain, particularly in the hippocampus, oxidative stress is strongly associated with the development of anxiety‐related behaviors (Bouayed et al. 2009; de Oliveira et al. 2007; Akudo and Idaguko 2023; Ghaffari‐Nasab et al. 2024). Research has shown that oxidative stress stimulates membrane lipid peroxidation and compromises membrane integrity. In addition, oxidative stress causes oxidation and dysfunction of cellular proteins and can even lead to DNA mutations. These cumulative effects contribute to the development of neurological diseases and anxiety disorders (Fedoce et al. 2018; Jaouad 2011). As a result, oxidative stress is recognized as a key factor in the pathogenesis of anxiety‐related behaviors and serves as a measurable indicator for tracking anxiety‐related behavior. Given that sleep disorders, such as insomnia, are among the most common complications of modern life, and given that anxiety is a significant consequence of insomnia—leading to reduced work performance and reduced quality of family life—we aimed to investigate potential interventions. Recognizing the anxiolytic and antidepressant properties of 4‐AP, this study was designed to investigate the effects of this drug on anxiety‐like behaviors induced by SD.

## 2 Materials and Methods

### 2.1 Animals

A group of 50 male BAL/c mice, 3 months old and weighing between 22 and 25 g, were obtained from the Urmia University Animal Care Center. The mice underwent a 1‐week acclimation period in the laboratory, where they were housed at 24 ± 2°C and exposed to a 12‐h light‐dark cycle (with lights off at 8 p.m. and on at 8 a.m.). During this period, the mice had free access to food and water. All procedures and experiments performed in this study followed the ethical guidelines developed by the Urmia University Ethics Committee for Animal Research, in line with the regulations set by the National Institutes of Health.

### 2.2 REM SD Protocol

**FIGURE 1:**
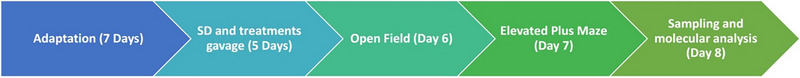
The flowchart of study progress.

The experimental protocol (Suchecki et al. 2000) consisted of placing 10 mice in a 42 × 30 × 20 cm Plexiglas tank equipped with 12 circular platforms. For the control group, wide platforms with a diameter of 6 cm were used to allow undisturbed sleep and to prevent mice from accidentally falling into the water. In the experimental groups, mice underwent a 5‐day (120 h) SD period using a modified multiple platform technique. The SD platforms were 1.5 cm in diameter and were placed with a 1 cm water layer below their upper surface. During this procedure, mice could move around the tank by jumping between the platforms. They were provided with unlimited access to water and food pellets, which were suspended from the cage cover. AP was purchased from Faran Chemical Company (Hamedan, Iran). The SD groups were divided into four subgroups: one group received normal saline (NS) and the rest received 4‐AP at doses of 0.25 mg/kg (AP‐0.25), 0.5 mg/kg (AP‐0.5), and 1 mg/kg (AP‐1). Both the drug and normal saline were administered daily by oral gavage at 9 a.m., coinciding with the SD period (Figure 1).

### 2.3 Open Field Tests (OFT)

The experimental set‐up consisted of a square arena made of black Plexiglas measuring 33 × 33 × 20 cm (purchased from Equip, Iran). The arena was divided into two peripheral and central zones. Each rat was introduced to the central zone, and the researchers recorded the total distance traveled as a measure of locomotor activity, as well as the time spent in the central zone over a 10‐min period. After each trial, the arena was thoroughly cleaned using a 70% ethanol solution to eliminate residual odors (Salehpour et al. 2019). All behavioral assessments were conducted between 8.5 a.m. and 1.5 p.m.

### 2.4 Elevated Plus‐Maze

The Elevated Plus Maze apparatus, manufactured by Equip in Iran, was used to assess anxiety. The apparatus is made of wooden material and has two open arms measuring 50 × 10 cm and two closed arms measuring 40 × 50 × 10 cm. There is also a central area measuring 10 × 10 cm. Before the start of the experiment, the animals were placed in the test room for 30 min. Then, they were placed in the central area of the maze for 5 min and allowed to move freely around the maze. Their behavior was recorded and analyzed using the EthoVision video tracking system. The percentage of time spent in the open arms (% OAT) and open arm entries (% OAE) were then calculated (Sanders et al. 2003).

### 2.5 Sampling

After behavioral testing, animals were anesthetized via intraperitoneal injection of ketamine hydrochloride (60 mg/kg) and xylazine (12 mg/kg). The heads of the mice were then removed using a guillotine, and their hippocampi were carefully dissected and immediately frozen at −80°C for further analysis.

### 2.6 Western Blot

Hippocampi were homogenized in cold suspension buffer using a homogenizer, and protein concentration was determined by the BCA protein assay. Proteins were then separated by SDS‐PAGE on a 10% gel and then transferred onto PVDF membranes (Millipore). The membranes were blocked with 3% BSA in PBS for 1 h before incubation overnight at 4°C with the following antibodies: anti‐AMPA‐R (Cell Signaling‐2460, diluted 1:1000), anti‐NMDA‐R (ab‐14596, Abcam:500F, diluted), (sc‐130349, diluted 1:500), and anti‐β‐actin (sc‐47778, diluted 1:500). For detection, secondary antibodies specific to the primary antibodies were used, including anti‐AMPA‐R (Sell Signaling, mouse monoclonal antibody), anti‐NMDA‐R (Abcam, rabbit antibody), anti‐TNF‐α (SANTA CRUZ, mouse monoclonal antibody), and anti‐β‐actin monoclonal antibody (SANTAe). β‐actin protein served as a housekeeping protein. ECL chemiluminescence detection was used to evaluate the blots, with the housekeeping protein detected on the same blot as the test proteins. Membranes and membrane processes were isolated and reprobed to allow for reuse, and the resulting blot images were analyzed using ImageJ software.

### 2.7 Assessment MDA and TAC

**FIGURE 2:**
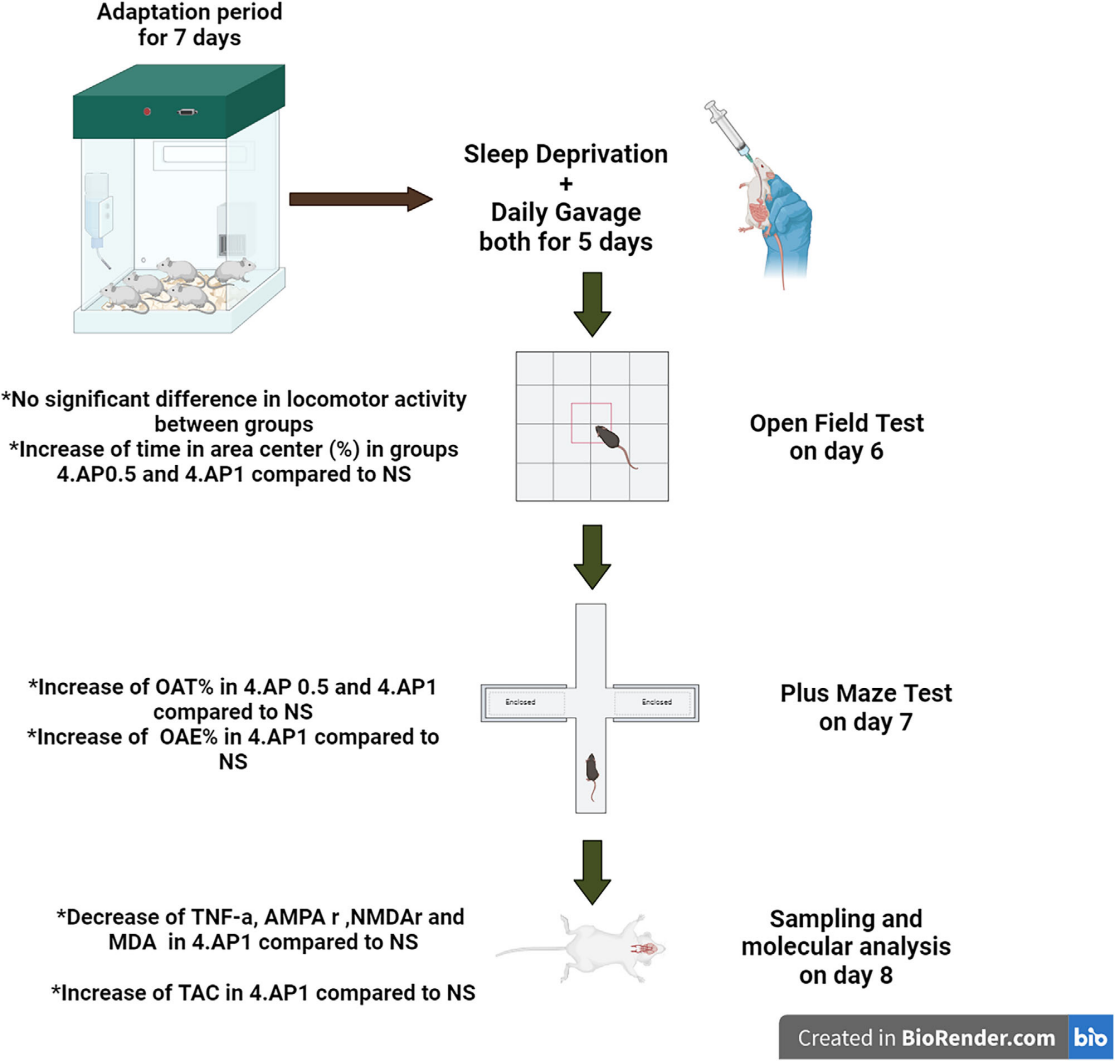
Flow chart of the study progress and the chief results obtained.

Hippocampal samples were homogenized in 1.15% KCl solution. The homogenate was then centrifuged at 1000 rpm for 10 min at 4°C. To estimate malondialdehyde (MDA) levels, the supernatant was analyzed using the thiobarbituric acid reactive substances (TBARS) assay. The absorbance of the reaction mixture was measured at 535 nm, and MDA levels in the brain were quantified and expressed as nanomoles per mg protein (Uchiyama and Mihara 1978). Total antioxidant capacity (TAC) was measured using the Randox Total Antioxidant Status Kit (Randox Laboratories Ltd, Crumlin, UK). This kit utilizes the decolorization of the radical cation 2,2'‐azinobis [3‐ethylbenzothiazoline‐6‐sulfonic acid] (ABTS•+) by antioxidants, with the extent of decolorization being proportional to the concentration and capacity of the antioxidants present. The color change was measured by measuring the absorbance at a wavelength of 600 nm (Figure 2 ).

**FIGURE 3:**
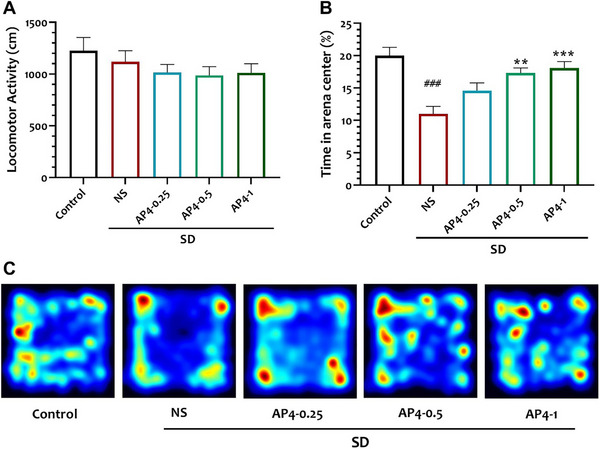
Effect of interventions on (A) locomotor activity and (B) time in the center area in study groups. Data are shown as mean ± SEM (*n* = 10 per group). (C) Corresponding heat maps show the combined traces of the mice from each group during the probe test of Open Field. One‐way ANOVA, followed by Tukey's post hoc test: **p < 0.01, ***p < 0.001 versus NS, ###*p* < 0.001 versus Control. There were no significant differences in activity levels.

**FIGURE 4:**
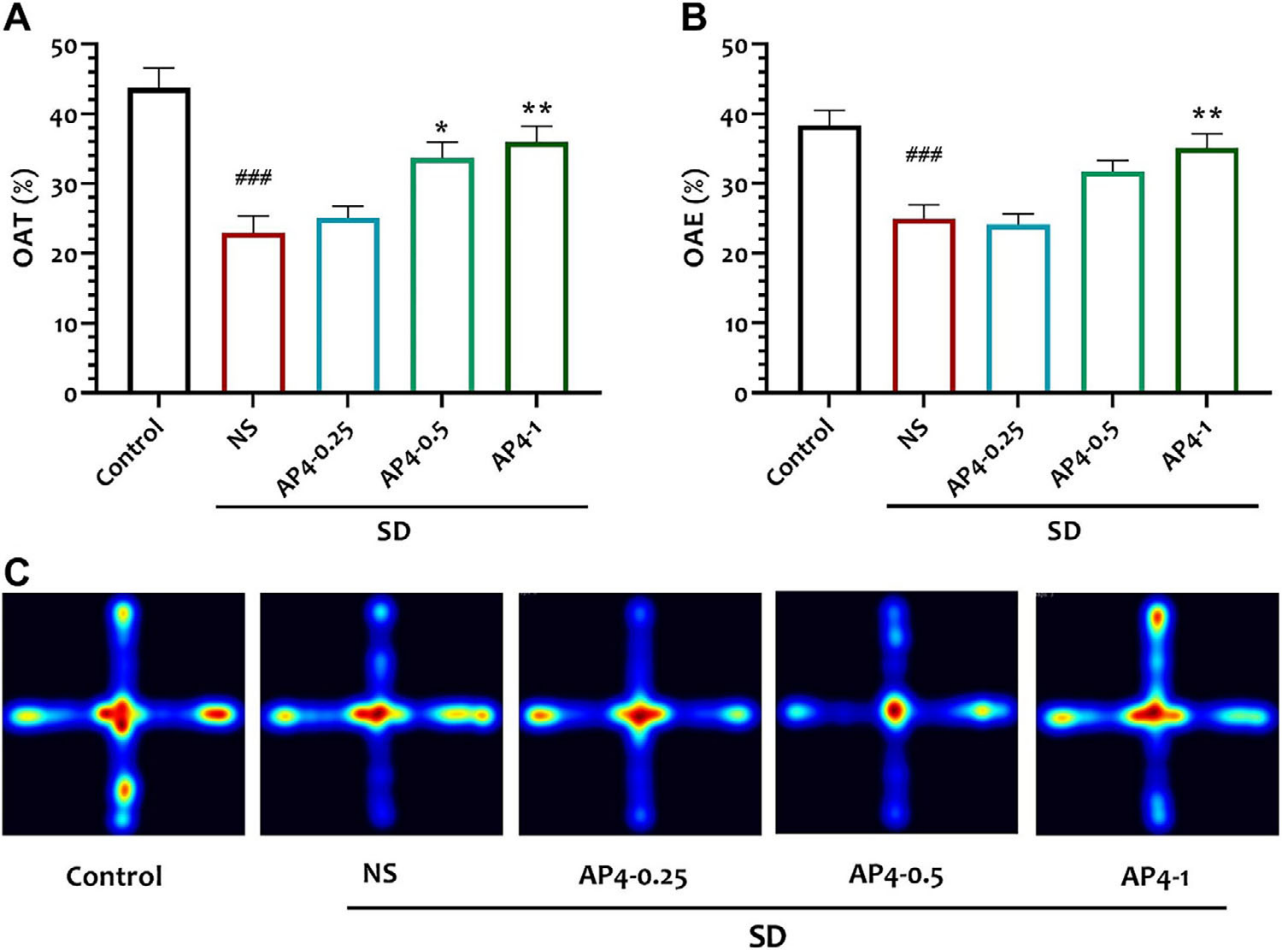
Effect of interventions on (A) percentage of open arm time and (B) percentage of open arm entrance. Data are shown as mean ± SEM (n = 10 per group). One‐way ANOVA, followed by Tukey's post hoc test: **p < 0.01, ***p < 0.001 versus NS, ###*p* < 0.001 versus Control. (C) Corresponding heat maps show the combined traces of the mice from each group during the probe test of Plus maze. The right and left branches represent open arms, and the upper and lower branches represent open arms.

**FIGURE 5:**
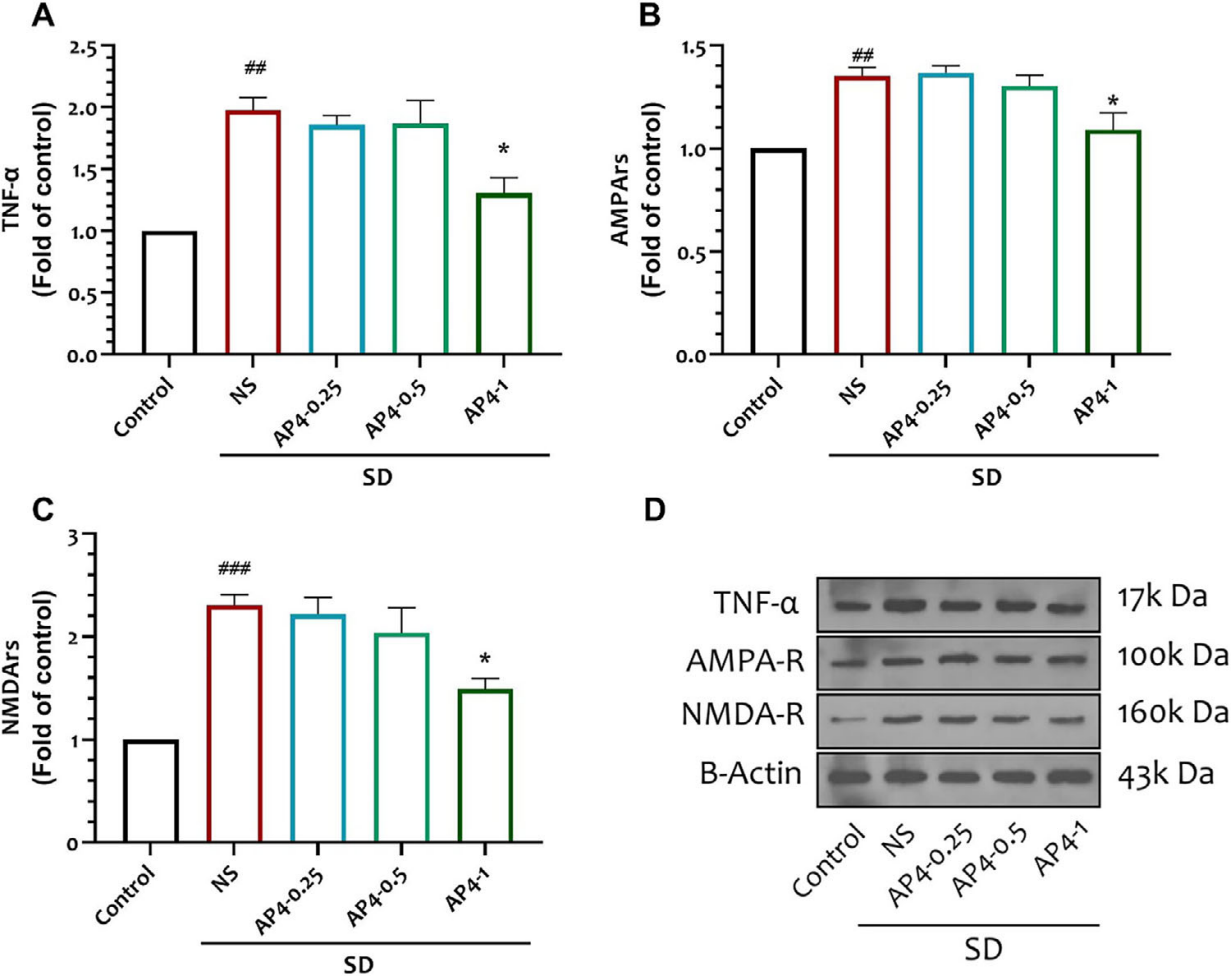
Effect of interventions on hippocampal (A) TNF‐α, (B) AMPA‐R, and (C) NMDA‐R in different study groups. (D) A representative immunoblotting image of proteins in different groups. Data are shown as mean ± SEM (*n* = 10 per group). One‐way ANOVA, followed by Tukey's post hoc test. *p < 0.05 versus NS, ###*p*<0.001 versus control group.

### 2.8 Statistical Analysis

The normality of the data distribution was confirmed by the Shapiro–Wilk test. All data are expressed as mean ± SEM. Data analysis was performed using SPSS 25 software, with one‐way analysis of variance (ANOVA) and Tukey's post hoc test. A significance level of *p* < 0.05 was considered statistically significant.

## 3 Results

Anxiety‐like behavior in animals was evaluated through the utilization of open field and elevated plus maze tests. In that way, the locomotor activity and time in the center area were measured by the open field test. The percentage of time elapsed in the open arm (OAT %) and percentage of open arm entrance (OAE %) were calculated by elevated plus‐maze test.

### 3.1 The Effect of AP‐4 and SD on Anxiety‐Like Behavior (Open Field Test)  

There was no statistically significant difference observed in locomotor activity (cm) among the groups as follows: Control compared to NS *p* = 0.9977, NS compared to AP4‐0.25 *p* = 0.9977, NS compared to AP4‐0.5 *p* = 0.6344, NS compared to AP4‐0.1 *p* = 0.7613 (*F* = 1.02, *N* = 10).

The NS group (11 ± 1.15) exhibited a significant decrease in the percentage of time spent in the center area compared to the control group (20 ± 1.29) (*p* < 0.0001). The percentage of time spent in the center area was found to have increased in both the AP‐0.5(17.3 ± 0.8) and AP‐1(18.1 + 0.97) groups when compared to the NS group (*p* < 0.01, *p* = 0.0019 and *p* < 0.001, *p* = 0.0004, respectively) (*F* = 1.02, *n* = 10) Fig 3.

### 3.2 The Effect of AP‐4 and SD on Anxiety‐Like Behavior (Elevated Plus Maze Test) 

The percentage of OAT and OAE in the NS group (22.97 ± 2.43, 24.95 ± 1.98, respectively) exhibited a significant decrease when compared to their control group (43.68 ± 2.86, 38.37 ± 2.09, respectively) (*p* < 0.0001). The OAT percentages in the AP‐0.5 (33.67 ± 2.26) and AP‐1 groups (35.98 ± 2.24) exhibited significant increases when compared to the NS group (22.97 ± 2.43) (*p* < 0.05, *p* = 0.022), and *p* < 0.01, *p* = 0.0028, respectively). Additionally, there was a significant increase in the percentage of OAE in the AP‐1 group (35.12 ± 2.04) compared to the NS group (24.95 ± 1.98) (*p* < 0.01, *p* = 0036) (*F* = 11.2, *n* = 10) Fig 4.

### 3.3 The Effect of 4‐AP and SD on TNF‐α

The levels of TNF‐α in the NS group (1.97 ± 0.1) demonstrated a significant increase in comparison to the control group, as indicated by a statistically significant difference (*p* < 0.01, *p* = 0.0012). The AP‐1 group (1.3 ± 0.1) demonstrated a statistically significant reduction in TNF‐α levels compared to the NS group (1.97 ± 0.1, *p* = 0.0188) (*p* < 0.05) (*F* = 14.02, *n* = 3) Fig 5A.

### 3.4 The Effect of 4‐AP and SD on AMPA Receptors

The NS group (1.35 ± 0.04) showed a statistically significant increase in the expression of AMPA‐R compared to the control group (*p* < 0.01, *p* = 0.005). The AP‐1 group (1.09 ± 0.08,) displayed a statistically significant decrease in the expression of AMPA‐R compared to the NS group (1.35 ± 0.04) (*p* < 0.05, *p* = 0.0363). (*F* = 11.39, *n* = 3) Fig 5B

### 3.5 The Effect of 4‐AP and SD on NMDA Receptors  

The NMDA‐R in the NS group (2.3 ± 0.1) exhibited a statistically significant elevation in comparison to the control group (*p* < 0.001, *p* = 0.0009). The AP‐1 group (1.49 ± 0.01) demonstrated a statistically significant decrease in the expression of NMDA‐R compared to the NS group (2.3 ± 0.1) (*p* < 0.05, *p* = 0.0281) (*F* = 14.14, *n* = 3) Fig 5C.

### 3.6 The Effect of 4‐AP and SD on Oxidative Stress 

**FIGURE 6:**
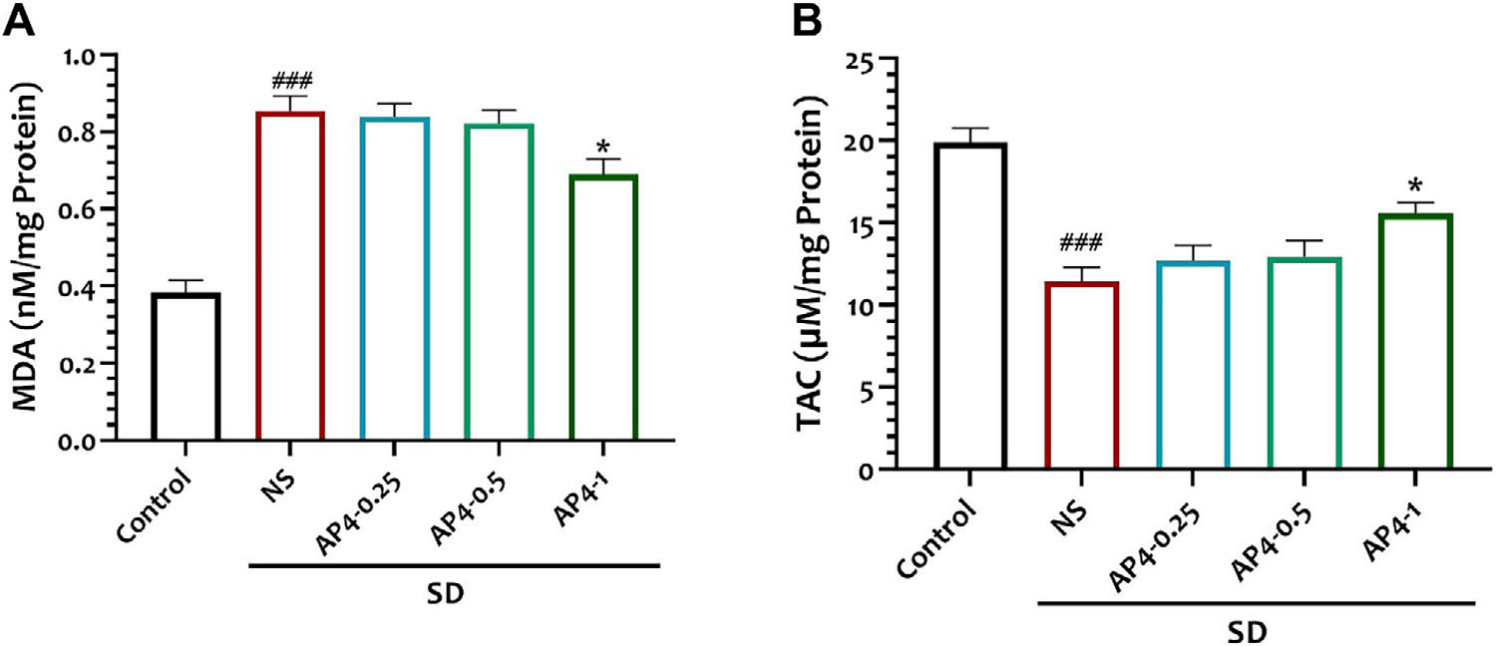
Effect of interventions on hippocampal (A) MDA and (B) TAC in study groups. Data are shown as mean ± SEM (*n* = 6 per group). One‐way ANOVA, followed by Tukey's post hoc test. *p < 0.05 versus NS, ###*p* < 0.001 versus control group. MDA, malondialdehyde.

The levels of MDA in the NS group (0.85 ± 0.03) were higher than those in the control group (0.38 ± 0.03) (*p* < 0.0001). The levels of MDA in the AP‐1 group (0.68 ± 0.04) exhibited a significant decrease when compared to the NS group (0.85 ± 0.03) (*p* < 0.05, *p* = 0.0329) (*F* = 30.31, *n* = 6) (Figure 6A)

The TAC in the NS group (11.44 ± 0.85) exhibited a significant decrease in comparison to the control group (19.89 ± 0.86) (*p* < 0.0001). The level of TAC in the AP‐1 group (15.58 ± 0.63) exhibited a significant increase when compared to the NS group (*p* < 0.05, *p* = 0.0235). (*F* = 15.07, *n* = 6) (Figure 6B).

## 4 Discussion

The research aimed to study the effects of 4‐AP on anxiety‐like behavior induced by SD, while examining the potential role of hippocampal TNF‐α, NMDA‐R, AMPA‐R, and oxidative stress markers (MDA and TAC) in this process. The results show that oral administration of 4‐AP can effectively reduce anxiety‐like behavior in SD rats. Currently, 4‐AP is widely used in the treatment of MS due to its ability to reduce MS‐related symptoms, such as impaired mobility and fatigue, as well as its role in restoring proper conduction of nerve impulses (Jensen et al. 2014; Tseng et al. 2016; Smits et al. 1994). The compound 4‐AP has been shown to improve nerve conduction and synaptic connectivity (Kasatkina 2016). Additionally, research suggests that 4‐AP has a positive effect on cognitive enhancement and may help reduce symptoms of depression (Arreola‐Mora et al. 2019; Rodriguez‐Leal et al. 2019). Increased anxiety levels have been widely documented as one of the most important consequences of SD. This has been well documented in meta‐analyses of human studies, which have shown that acute SD leads to anxiety‐like behaviors (Pires et al. 2016). Furthermore, several studies highlight this connection, as sleep‐deprived mice also exhibit anxiety‐like behavior (Gonzalez‐Castañeda et al. 2016; Wang et al. 2021; Vollert et al. 2011; Tai et al. 2020; Liu et al. 2022; Silva et al. 2004). SD is associated with increased cortisol levels (Wright et al. 2015), indicating dysregulation of the hypothalamic‐pituitary‐adrenal (HPA) axis. This dysregulation results in increased levels of glucocorticoid hormones such as cortisol, which can have significant adverse psychological effects, including anxiety (Hirotsu et al. 2015). One possible explanation for this is that SD is associated with hyperactivity of the HPA axis (Spiegel et al. 1999). Furthermore, short‐term SD has been reported to increase glutamate concentrations in the basal ganglia (Korenic et al. 2020), and glutamatergic neurotransmission in this brain region has been implicated in mood disorders, including anxiety (Pagonabarraga et al. 2021).

Because the effect of REM SD on anxiety‐like behaviors in rodents has not been relatively studied, our research focused on examining the relationship between REM SD and insomnia and anxiety. In addition, we examined the effect of 4‐AP on this relationship. Anxiety‐like behavior, characterized by intense fear and distress, is associated with REM SD (Mathangi et al. 2012). Findings from studies of REM and total SD in rodents consistently demonstrate the anxiogenic effects of the disorder (Silva et al. 2004; Suchecki et al. 2002; Suchecki et al. 2002). Based on current research, studies have shown that the presence of NMDA receptors in the amygdala and cerebral cortex is associated with anxiety through glutamatergic neurotransmission (Wang et al. 2022). Furthermore, the literature strongly emphasizes the significant contribution of hippocampal NMDA receptors in the initiation of anxiety (Wang et al. 2022; Barkus et al. 2010; Ghasemi et al. 2014; Salimando et al. 2020). Furthermore, AMPA‐Rs have been shown to influence the development of anxiety‐like behaviors (da Cunha et al. 2008; Kapus et al. 2008). The observed increase in NMDA‐R and AMPA‐R levels in animals subjected to SD, followed by a decrease in these receptor levels after 4‐AP treatment, may indicate the involvement of compensatory mechanisms in the SD‐induced anxiety response. Notably, several drugs that act as NMDA‐R antagonists have potent anxiolytic effects (Porter et al. 1989; Wiley et al. 1995; Corbett and Dunn 1993; Bertoglio and Carobrez 2003). In particular, structural defects in NMDA‐Rs in the hippocampus have been shown to reduce anxiety in rodents (Barkus et al. 2010). Similarly, in the case of AMPA‐Rs, research has shown that the administration of an AMPA‐R antagonist into the hippocampus can have profound anxiolytic effects in mice (Kapus et al. 2008). Many other studies support the idea that blocking AMPA‐Rs can lead to anxiolytic effects (Alt et al. 2006; Bi et al. 2013; Kotlinska and Liljequist 1998).

As previously mentioned, TNF‐α has the ability to activate NMDA‐R and AMPA‐R receptors in the brain (Olmos and Lladó 2014; Jara et al. 2007). However, in the present study, it was observed that the expression of TNF‐α, which normally acts as an agonist of NMDA‐R and AMPA‐R, was reduced after treatment with 4‐AP. Interestingly, increased levels of TNF‐α in the hippocampus itself have been associated with anxiety‐like behaviors (Kemp et al. 2022; Haji et al. 2012), and there is strong evidence that reduced hippocampal TNF‐α mRNA expression is associated with reduced anxiety‐like behaviors in mice (Fourrier et al. 2019). In addition, a significant decrease in MDA production and an increase in TAC concentration were observed in the 4‐AP‐1 group. This suggests that 4‐AP may have the potential to reduce oxidative stress. It is important to emphasize that oxidative stress in the hippocampus has been associated with the onset of anxiety‐like behaviors (Ghaffari‐Nasab et al. 2024; de Oliveira et al. 2007). Our results are consistent with the effects of fluoxetine, an SSRI, which exhibits anxiolytic properties in mice by reducing oxidative stress and increasing antioxidant capacity in the hippocampus (Battal et al. 2014). Furthermore, venlafaxine, a widely used antidepressant, has been shown to reduce oxidative stress (Abdel‐Wahab and Salama 2011) and simultaneously reduce anxiety‐like behaviors in animal and human studies (Lapmanee et al. 2013, 2012; Gorman and Papp 2000; Katzman and Jacobs 2007). A similar effect has been observed with various antioxidants (Ienco et al. 2011; Ramos et al. 2016). It should be acknowledged that one of the limitations of the current study is its focus on male mice, and the findings would have been stronger if female mice had been included in the study. Future studies on the behavioral effects of 4‐AP are recommended to consider both male and female animals for a more comprehensive understanding. Additionally, this study did not investigate the expression levels of AMPAR and NMDAR in other brain regions, nor did it analyze other anxiety‐related molecules like GABA receptors and serotonin receptors. Moreover, the potential side effects of 4‐AP on healthy mice have yet to be explored. It is important to highlight that this study serves as a precursor to encourage further research into the potential mechanisms underlying the results obtained from the current study. It is suggested that these crucial matters be taken into account in future studies.

It can be suggested that a potential mechanism underlying the reduction of anxiety‐like behaviors due to REM SD by 4‐AP involves a reduction in the levels of NMDA‐R and AMPA‐R receptors in the hippocampus, which are closely associated with the initiation of anxiety‐related behaviors. In addition, this reduction may be attributed to a reduction in the amount of TNF‐α, a molecule that directly stimulates NMDA‐R and AMPA‐R receptors. Additionally, 4‐AP could potentially reduce anxiety‐like behaviors by reducing oxidative stress induced by REM SD in the hippocampus. This is important because oxidative stress in the hippocampus is a known contributor to anxiety‐like behavior.

There is no clinical study on the effect of 4‐AP on anxiety behavior induced by insomnia. Experimentally, clinical trials assessing the efficacy and safety of low‐dose 4‐AP in patients with insomnia‐related anxiety are necessary. Potential clinical studies could begin with pilot trials assessing the acute effects of 4‐AP on sleep‐deprived individuals using anxiety scales, EEG, and cortisol measurements as biomarkers. Randomized controlled trials (RCTs) should then evaluate the long‐term anxiolytic effects of 4‐AP compared to existing treatments, assessing parameters such as sleep quality, heart rate variability (HRV), and functional MRI connectivity changes in anxiety‐related brain circuits. Additionally, investigating dose‐response relationships and potential adverse effects will be crucial to determining its therapeutic window. Further multi‐brain region analysis using techniques like fMRI and optogenetics could provide insights into the broader neural circuits affected by 4‐AP. Investigating its effects on GABAergic and serotonergic systems would further clarify its role in anxiety regulation.

Given its ability to modulate glutamatergic transmission, reduce neuro‐inflammation, and mitigate oxidative stress, 4‐AP may hold promise as a novel therapeutic strategy for anxiety disorders associated with SD, warranting further investigation.

## Author Contributions

**Ehsan Hosseini**: investigation, writing–review and editing, visualization, methodology, validation, software, formal analysis, data curation, supervision, writing–original draft, conceptualization, resources, project administration.

## Ethics Statement

The procedures and experiments conducted in this study adhered to the regulations set forth by the Urmia University Ethical Committee for the protection of animals, in accordance with the guidelines established by the National Institute of Health.

## Conflicts of Interest

The authors declare no conflicts of interest.

### Peer Review

The peer review history for this article is available at https://publons.com/publon/10.1002/brb3.70382.

## Data Availability

The data that support the findings of this study are available from the corresponding author upon reasonable request.
